# Assessment of enrollment characteristics for Children’s Oncology Group (COG) upfront therapeutic clinical trials 2004-2015

**DOI:** 10.1371/journal.pone.0230824

**Published:** 2020-04-23

**Authors:** Kelly E. Faulk, Amy Anderson-Mellies, Myles Cockburn, Adam L. Green

**Affiliations:** 1 Center for Cancer and Blood Disorders, Department of Pediatrics, School of Medicine and Children’s Hospital Colorado, University of Colorado Aurora, CO, United States of America; 2 University of Colorado Comprehensive Cancer Center, Aurora, CO, United States of America; Cincinnati Children’s Hospital Medical Center, UNITED STATES

## Abstract

**Background:**

Improvements in pediatric cancer survival are attributed to cooperative clinical trials. Under-representation of specific demographic groups has been described in adult and pediatric cancer trials and poses a threat to the generalizability of results. An evaluation of data provided by the Children’s Oncology Group (COG) of upfront trial enrollment for US patients 0 to 29 years old between 2004 and 2015 was performed.

**Methods:**

US cancer cases were estimated using incidence data and US population estimates from the Surveillance, Epidemiology, and End Results Program and compared to observed COG cases. Percent enrollment and standardized ratios of enrollment were calculated across demographic, disease, and socioeconomic groups. The COG website was utilized to quantify available trials and assess age eligibility.

**Results:**

19.9% of estimated US cancer patients age 0 to 19 years enrolled on COG trials. Younger patients were more represented across diseases and races/ethnicities. Patients with hematologic malignancies were more represented compared to solid and central nervous system (CNS) tumors.

**Conclusion:**

COG trial enrollment rates are declining when compared to previously published data, potentially from challenges in pediatric drug development, difficulty designing feasible trials for highly curable diagnoses, and issues ensuring trial availability for the heterogeneous group of solid and CNS tumors. Though racial/ethnic groups and county-level socioeconomic factors were proportionally represented, under representation of the adolescent/young adult (AYA) population and younger patients with solid and CNS tumors remains a concern. Targeted efforts should focus on these subgroups and further research should evaluate AYA enrollment rates across all available trials.

## Introduction

The improvement in childhood cancer mortality over several decades [1, 2] is attributed to treatment advances from cooperative clinical trials across the United States (US). Compared to adult cancer patients, 1.5–4% of whom enroll, [3, 4] trial participation for young cancer patients is reported to be much higher, with enrollment rates of 27–86%. [2, 3, 5–10] Underrepresentation of racial/ethnic minorities in trials has been consistently reported in adult cancer populations, and similar disparities in pediatric and adolescent enrollment have been published with regard to age, race/ethnicity, and cancer diagnosis. [6–9, 11–14] However, previous studies were commonly single institution, were performed decades ago, and have typically focused on adult National Cancer Institute (NCI) trial enrollment; thus, a comprehensive, modern evaluation of pediatric and young adult trial enrollment is needed.

The Children’s Oncology Group (COG) was created in 2000 as a merger of four cooperative groups. With over 200 institutions, it represents the largest pediatric oncology cooperative group in the world, with the majority of children diagnosed with cancer in the US cared for at COG institutions. [5] Given the large scope of COG clinical research, there is a necessary emphasis on equal access to trials and proportional representation of enrolled patients to ensure generalizability of results. Lund *et al*. performed an evaluation of COG enrollment between 2000–2003 for US children age 0 to 19 years old, comparing observed proportions of children enrolled in COG therapeutic trials to estimated proportions of cancer cases based on data from the Surveillance, Epidemiology, and End Results (SEER) Program. Despite relatively proportional representation of racial/ethnic groups, the analysis highlighted underrepresentation of adolescents, regardless of diagnosis. [9]

We performed an updated assessment of pediatric and young adult COG trial participation between 2004 and 2015 using similar but expanded methodology, surveying for disparities in enrollment to identify possible barriers. We expanded the age to include young adult patients, who are identified as a key population affected by health disparities. [15] Further, we assessed enrollment by specific cancer diagnoses as well as county-level socioeconomic factors to provide a comprehensive view of COG trial enrollment.

## Methods

### Data sources and cohort creation

Four data sources were used: COG enrollment data (provided by the COG data center), SEER 18 incidence data, [16] US population estimates (obtained through SEER), [17] and sociodemographic information from the American Community Survey (ACS). [18] We chose to utilize the SEER database given its representativeness of the overall study population, its ability to assess area-based socioeconomic data, and the allowance of easy comparison to prior studies performed which also used SEER. Of note, SEER 18 registries represent approximately 28% of the US population based on the 2010 Census. [19] A SEER cohort was created composed of patients age 0 to 29 years diagnosed with a malignancy between 2004 and 2015, selecting the first matching record for each person. Disease classification was determined using the International Classification of Childhood Cancer (ICCC), with lymphoid leukemia and non-Hodgkin lymphoma further delineated through histology codes. All malignancies were included and diagnoses were broadly categorized into hematologic (ICCC site groups I and II), CNS (III and Xa), solid (IV-IX, Xb-Xe, and XI), and unclassified (XII and unclassified). Age was classified into five-year age groups (we defined the pediatric age group as 0–14 years old and the adolescent/young adult (AYA) as 15–29 years old), and race/ethnicity was categorized as Hispanic (all races) and non-Hispanic, which were subcategorized into White, Black, Asian/Pacific Islander, and American Indian/Alaskan. Non-Hispanic patients with unknown race were excluded (n = 1,945) based on not having a corresponding population estimate from which to calculate incidence. Next, a de-identified dataset from COG was obtained including US patients 0 to 29 years enrolled onto upfront (i.e. newly diagnosed disease) therapeutic trials (regardless of phase) between 2004 and 2015. While we did not specifically limit trials by availability (COG groupwide versus limited site), most trials included were open groupwide. International enrollments outside of the US were not included. Patients enrolled on multiple trials were only considered for the first trial to which they enrolled. Subjects whose age at enrollment or gender was unknown were excluded (n = 61). Patients were classified by disease type using the malignancy prompting trial enrollment. We prioritized classification by histology rather than disease site, given that histology typically determined trial eligibility. When able, we used trial eligibility to clarify diagnosis. Our final study populations included 114,316 SEER patients and 36,683 COG patients.

Given that patient-level socioeconomic data does not exist in COG or SEER, county-level attributes were used to ascertain the socioeconomic characteristics of the area where patients lived at the time of COG registration. While patient county is readily available in SEER data, the COG data included only a patient zip code. The 2010 Zip Code Tabulation Area (ZCTA) to County Relationship File from the US Census Bureau was used to translate COG patient zip code to county. In cases where a ZCTA crossed county boundaries, patients were assigned to the county containing the largest percentage of the ZCTA population. County could not be established for 520 COG patients (1.2%) and was unknown for 34 patients in SEER (<1%). All US counties were classified into quintiles using data from ACS 2010–2014 and categorized using the highest or lowest two quintiles, as applicable, for low education attainment (≥ 15.6% individuals aged 25 or older with less than a high school education); high poverty (≥ 17.7% individuals with income below poverty); high percentage foreign-born (≥ 3.5% individuals born outside of the US); and low household income (≤ $42,300 median household income). Individuals with unknown county were noted as missing for each socioeconomic factor.

### Calculation of estimated US cancer cases

**Fig 1:**
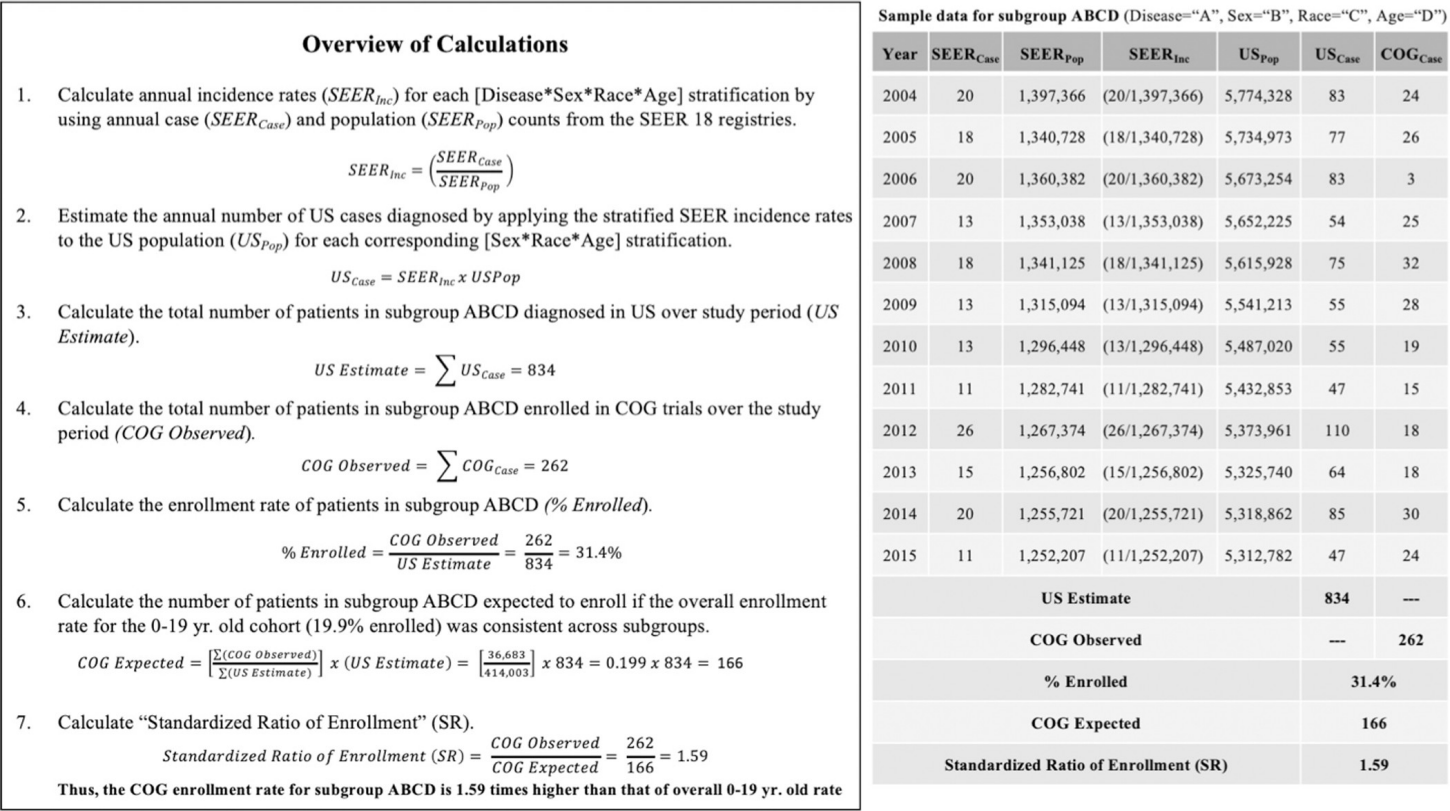
Calculation of standardized ratio of enrollment (SR) for COG subgroups.

A schema of study calculations is depicted in Fig 1. Annual incidence rates were calculated for each tumor type and stratified by age, gender, and race/ethnicity using SEER*Stat software (version 8.3.5). Annual US population estimates stratified by age, gender, and race/ethnicity were also generated using SEER*Stat software. The estimated number of US cases for each stratification was calculated by multiplying the SEER incidence rate by the corresponding US population estimate, with the sum of all the stratifications representing the estimated number of cancer cases diagnosed in the US over the entire study period. For each of the four socioeconomic factors, annual incidence rates and US population estimates were generated as previously described with the additional stratification of the county-based socioeconomic factor. The resulting total estimates of US cases varied across socioeconomic factors, and each differed slightly from the overall estimate of US cases obtained from the initial stratifications by tumor type, age, gender, and race/ethnicity. To account for this, the US estimates for each socioeconomic factor were converted to proportions (e.g. percent high poverty and percent not high poverty) and multiplied by the overall initial estimate of US cases.

### Calculation of enrollment ratios

Observed COG cases were tabulated for each subgroup from the COG dataset. Enrollment percentage was calculated by dividing the observed number of COG cases by the corresponding estimate of US cases. We chose to standardize all calculations to the enrollment percentage of one identified patient group to provide easier comparison across subgroups, and the 0 to 19-year age group was deemed to be the most representative of the overall COG cohort. Thus, the enrollment percentage for patients 0 to 19-years old (19.9%) was multiplied by the estimate of US cases for each subgroup to calculate the expected number of COG cases. COG observed cases were then divided by COG expected cases to calculate a Standardized Ratio of enrollment (SR). A SR of >1 or <1 indicates COG enrollment was higher or lower than expected, respectively. 95% confidence intervals for SR were calculated using effect size +/- 1.96 multiplied by the standard error of the effect size.

### Assessment of available trials

A search of upfront trials was performed on the COG member website, with access granted by COG. Trials were included if the “open to accrual” and “study closed” dates occurred at any time within 2004 to 2015. To achieve the broadest evaluation of available trials for newly diagnosed patients, we excluded trials in which eligibility required a specific cytogenetic abnormality (namely, Ph+ ALL). We chose to limit our evaluation to larger diagnostic categories and opted to not display available trials specific to smaller subgroups such as infant ALL, Down Syndrome leukemia, juvenile myelomonocytic leukemia (JMML), acute promyelocytic leukemia (APML), and MDS. Eligibility criteria of trials were evaluated to determine the upper age limit permitted.

## Results

### Study cohorts

Between 2004 and 2015, extrapolating from SEER data, there were 414,003 cancer diagnoses in patients 0 to 29 years old in the US (Table 1). Hematologic malignancies accounted for 29%, solid tumors 59%, central nervous system (CNS) tumors 11%, and unclassified disease 1%. Genders were equally represented. Whites comprised 66%, followed by Hispanics at 18%. Patients in older age groups had the highest proportion of cancers, with 35% in patients 25 to 29 years old, and 23% in those 20 to 24 years. Among children, 15 to 19-year-olds had the highest proportion at 14%, followed by 0 to 4-year-olds at 13%. For socioeconomic factors, 35% of cases were from a high poverty county, 82% from a county with a high foreign-born population, 29% from a low education attainment county, and 14% from a low household income county.

**Table 1:** Estimated US cancer cases by SEER and observed COG enrollment, 2004–2015.

Demographic Factor	US Estimated by SEER		COG Observed		COG Observed/US Estimated = % Enrolled
	*No*.	*%* ^a^	*No*.	*%* ^a^	
**Total, 0 to 19 years**	**174,317**	**42**	**34,759**	**95**	**19.9%**
**Total, 0 to 29 years**	**414,003**	**100**	**36,683**	**100**	**8.9%**
**Disease Type**					
Hematologic malignancy	121,396	29	25,144	68	20.7%
Solid tumor	243,253	59	8,974	24	3.7%
CNS tumor	46,338	11	2,565	7	5.5%
Unclassified	3,016	1	0	- - -	- - -
**Gender**					
Male	201,967	49	20,457	56	10.1%
Female	212,036	51	16,226	44	7.7%
**Race/Ethnicity**					
White	272,336	66	21,680	59	8.0%
Black	45,112	11	3,684	10	8.2%
Hispanic	75,494	18	7,808	21	10.3%
Asian/Pacific Islander	17,469	4	1,412	4	8.1%
American Indian, Alaskan	3,592	1	170	<1	4.7%
2+ races or Unknown ^b^	0	- - -	1,929	5	- - -
**Age (years)**					
0–4	53,011	13	14,367	39	27.1%
5–9	29,839	7	8,673	24	29.1%
10–14	34,266	8	5,488	15	16.0%
15–19	57,201	14	6,231	17	10.9%
20–24	94,601	23	1,680	5	1.8%
25–29	145,085	35	244	<1	0.2%
**Year of Enrollment**					
2004–2006	97,685	24	8,427	23	8.6%
2007–2009	103,811	25	10,312	28	9.9%
2010–2012	104,871	25	9,094	25	8.7%
2013–2015	107,636	26	8,850	24	8.2%
**Socioeconomic factors** ^c^					
High poverty county	143,351	35	12,379	34	8.6%
Not high poverty county	270,652	65	23,873	65	8.8%
High foreign-born county	340,780	82	29,595	81	8.7%
Not high foreign-born county	73,223	18	6,657	18	9.1%
Low education attainment county	118,746	29	10,955	30	9.2%
Not low education attainment county	295,257	71	25,297	67	8.6%
Low household income county	55,906	14	4,740	13	8.5%
Not low household income county	358,097	86	31,512	86	8.8%

^a^ Percentages may not sum to 100 due to rounding.

^b^ All patients in SEER were categorized into a primary race/ethnicity, whereas COG had patients with unknown race/ethnicity (n = 2,232) or those classified as multiple races (n = 10).

^c^ High poverty defined as ≥ 17.7% individuals with income below poverty); high foreign-born defined as ≥ 3.5% individuals born outside of the US; low education attainment defined as ≥ 15.6% individuals aged 25 or older with less than a high school education; and low household income defined as ≤ $42,300 median household income.

Abbreviations: SEER, Surveillance, Epidemiology, and End Results Program; COG, Children’s Oncology Group; CNS, central nervous system.

The number of patients enrolled in upfront COG trials during the same period was 36,683. Males comprised more of the enrolled population than females (56% versus 44%), and Whites accounted for 59%, followed by Hispanics at 21%. The COG cohort was largely young: 0 to 4-year-olds (39%), 5 to 9-year-olds (24%), 15 to 19-year-olds (17%) and 10 to 14-year-olds (15%), versus 20 to 24-year-olds (5%) and 25 to 29-year-olds (<1%). Enrollment initially increased from 2004–2006 to 2007–2009, but subsequently declined during the 2010–2012 and 2013–2015 time periods. Socioeconomic factors mirrored data from SEER, with 81% from a county with a high foreign-born population, 34% from a high poverty county, 30% from a low education attainment county, and 13% from a low household income county.

### Enrollment by major demographic factors

An assessment of COG enrollment by major demographic factors and stratified by age is displayed in Table 2. Enrollment rates declined with rising age group. Males and females enrolled fairly equally across all age groups, though a slight overrepresentation of males was present among older groups. Though racial/ethnic groups showed fairly equivalent representation overall, American Indian/Alaskan patients were noted to be enrolled relatively less than expected across all age groups. Patients with hematologic malignancies were consistently more represented than solid or CNS tumors. Socioeconomic factors showed grossly equivalent representation within each age group.

**Table 2:** COG enrollment by major demographic factors, stratified by age, 2004–2015.

Age	Demographic Factor	US Estimated by SEER ^a^	COG Observed/US Estimated = % Enrolled	COG Expected (E)	COG Observed (O)	COG O/E = SR ^b^ (95% CI)
**0 to 9 years**	**Total 0 to 9 years**	**82,850**	**27.8%**	**16,487**	**23,039**	**1.40 (1.38, 1.41)**
	**Gender**					
	Male	44,732 (54%)	28.0%	8,902	12,544	1.41 (1.38, 1.43)
	Female	38,118 (46%)	27.5%	7,585	10,495	1.38 (1.36, 1.41)
	**Race/Ethnicity** ^c^					
	White	48,923 (59%)	27.7%	9,736	13,543	1.39 (1.37, 1.41)
	Black	10,004 (12%)	21.4%	1,991	2,140	1.07 (1.03, 1.12)
	Hispanic	19,330 (23%)	25.9%	3,847	5,007	1.30 (1.27, 1.34)
	Asian/Pacific Islander	3,797 (5%)	24.1%	756	916	1.21 (1.13, 1.29)
	American Indian, Alaskan	796 (1%)	14.7%	158	117	0.74 (0.60, 0.87)
	**Disease type**					
	Hematologic malignancy	34,954 (42%)	45.7%	6,956	15,957	2.29 (2.26, 2.33)
	Solid tumor	29,009 (35%)	19.6%	5,773	5,682	0.98 (0.96, 1.01)
	CNS tumor	18,538 (22%)	7.6%	3,689	1,400	0.38 (0.36, 0.40)
	**Socioeconomic factors** ^d^					
	High poverty county	28,659 (35%)	27.0%	5,703	7,733	1.36 (1.33, 1.39)
	Not high poverty county	54,191 (65%)	27.8%	10,784	15,057	1.40 (1.37, 1.39)
	High foreign-born county	68,236 (82%)	27.4%	13,579	18,700	1.38 (1.36, 1.40)
	Not high foreign-born county	14,614 (18%)	28.0%	2,908	4,090	1.41 (1.36, 1.45)
	Low education attainment county	25,365 (31%)	27.0%	5,048	6,860	1.36 (1.33, 1.39)
	Not low education attainment county	57,485 (69%)	27.7%	11,439	15,930	1.39 (1.37, 1.41)
	Low household income county	11,435 (14%)	25.6%	2,275	2,922	1.28 (1.24, 1.33)
	Not low household income county	71,415 (86%)	27.8%	14,212	19,868	1.40 (1.38, 1.42)
**10–19 years**	**Total 10 to 19 years**	**91,467**	**12.8%**	**18,202**	**11,719**	**0.64 (0.63, 0.65)**
	**Gender**					
	Male	48,444 (53%)	13.8%	9,640	6,695	0.69 (0.68, 0.71)
	Female	43,023 (47%)	11.7%	8,562	5,024	0.59 (0.57, 0.60)
	**Race/Ethnicity** ^c^					
	White	58,193 (64%)	11.9%	11,580	6,926	0.60 (0.58, 0.61)
	Black	10,862 (12%)	12.4%	2,162	1,344	0.62 (0.59, 0.65)
	Hispanic	17,889 (20%)	13.7%	3,560	2,457	0.69 (0.66, 0.72)
	Asian/Pacific Islander	3,740 (4%)	11.3%	744	422	0.57 (0.51, 0.62)
	American Indian, Alaskan	783 (1%)	5.9%	156	46	0.30 (0.21, 0.38)
	**Tumor Type**					
	Hematologic malignancy	34,878 (38%)	22.9%	6,941	8,004	1.15 (1.13, 1.18)
	Solid tumor	42,336 (46%)	6.3%	8,425	2,681	0.32 (0.31, 0.33)
	CNS tumor	13,754 (15%)	7.5%	2,737	1,034	0.38 (0.35, 0.40)
	**Socioeconomic factors** ^d^					
	High poverty county	31,076 (34%)	12.9%	6,184	4,022	0.65 (0.63, 0.67)
	Not high poverty county	60,391 (66%)	12.5%	12,018	7,537	0.63 (0.61, 0.64)
	High foreign-born county	74,473 (81%)	12.6%	14,820	9,347	0.63 (0.62, 0.64)
	Not high foreign-born county	16,994 (19%)	13.0%	3,382	2,212	0.65 (0.63, 0.68)
	Low education attainment county	26,904 (29%	13.3%	5,354	3,583	0.67 (0.65, 0.69)
	Not low education attainment county	64,563 (71%)	12.4%	12,848	7,976	0.62 (0.61, 0.63)
	Low household income county	12,922 (14%)	12.3%	2,572	1,593	0.62 (0.59, 0.65)
	Not low household income county	78,545 (86%)	12.7%	15,630	9,966	0.64 (0.62, 0.65)
**20–29 years**	**Total 20 to 29 years**	**239,686**	**0.8%**	**47,698**	**1,924**	**0.04 (0.04, 0.04)**
	**Gender**					
	Male	108,791 (45%)	1.1%	21,649	1,218	0.06 (0.05, 0.06)
	Female	130,895 (55%)	0.5%	26,048	706	0.03 (0.02, 0.03)
	**Race/Ethnicity** ^c^					
	White	165,220 (69%)	0.7%	32,879	1,211	0.04 (0.03, 0.04)
	Black	24,246 (10%)	0.8%	4,825	199	0.04 (0.03, 0.05)
	Hispanic	38,275 (16%)	0.9%	7,617	344	0.05 (0.04, 0.05)
	Asian/Pacific Islander	9,932 (4%)	0.7%	1,976	74	0.04 (0.03, 0.05)
	American Indian, Alaskan	2,013 (1%)	0.3%	401	7	0.02 (0.00, 0.03)
	**Disease type**					
	Hematologic malignancies	51,564 (21%)	2.3%	10,261	1,185	0.12 (0.11, 0.12)
	Solid tumors	171,908 (72%)	0.4%	34,210	609	0.02 (0.02, 0.02)
	CNS tumors	14,046 (6%)	0.9%	2,795	130	0.05 (0.04, 0.05)
	**Socioeconomic factors** ^d^					
	High poverty county	83,616 (35%)	0.7%	16,640	622	0.04 (0.03, 0.04)
	Not high poverty county	156,070 (65%)	0.8%	31,058	1,277	0.04 (0.04, 0.04)
	High foreign-born county	198,068 (83%)	0.8%	39,416	1,546	0.04 (0.04, 0.04)
	Not high foreign-born county	41,618 (17%)	0.8%	8,282	353	0.04 (0.04, 0.05)
	Low education attainment county	66,487 (28%)	0.8%	13,231	510	0.04 (0.03, 0.04)
	Not low education attainment county	173,199 (72%)	0.8%	34,467	1,389	0.04 (0.04, 0.04)
	Low household income county	31,548 (13%)	0.7%	6,278	223	0.04 (0.03, 0.04)
	Not low household income county	208,138 (87%)	0.8%	41,419	1,676	0.04 (0.04, 0.04)

^a^ Percentages may not sum to 100 due to rounding.

^b^ Using total percent enrollment of patients 0 to 19 years old (19.9%).

^c^2+ races and unknown race/ethnicity patients were excluded given no corresponding US SEER data available.

^d^ High poverty defined as ≥ 17.7% individuals with income below poverty); high foreign-born defined as ≥ 3.5% individuals born outside of the US; low education attainment defined as ≥ 15.6% individuals aged 25 or older with less than a high school education; and low household income defined as ≤ $42,300 median household income.

Higher enrollment than expected by SEER.

Lower enrollment than expected by SEER.

Abbreviations: SR, Standardized Ratio of Enrollment; CI, Confidence Interval; CNS, Central Nervous System; SEER, Surveillance, Epidemiology, and End Results.

### Enrollment by disease type

Enrollment was evaluated by disease type and stratified by age, using the most prevalent diagnoses for each age group within the COG cohort (Table 3). Within hematologic malignancies, patients with acute lymphoblastic leukemia/lymphoma (ALL/LyL) had higher enrollment, and those with Hodgkin lymphoma had lower enrollment than expected across all age groups. Patients with acute myeloid leukemia/myelodysplastic syndrome (AML/MDS) showed increased enrollment from expected only among patients 0 to 19 years old. Within solid and CNS tumors, patients with soft tissue sarcoma (STS) and glioma had reduced enrollment from expected across all age groups. Additionally, the youngest patients (0 to 9 years) with medulloblastoma (MBL) had reduced enrollment from expected, though enrollment for those age 10 to 19 years was increased.

**Table 3:** COG enrollment by disease type (most prevalent in age group), stratified by age, 2004–2015.

Age	Disease Type	US Estimated by SEER ^a^	COG Observed/ US Estimated = % Enrolled	COG Expected (E)	COG Observed (O)	COG O/E = SR ^b^ (95% CI)
**0–9 years**	**Hematologic malignancies**					
	*ALL & LyL*	25,154 (30%)	56.6%	5,006	14,236	2.84 (2.80, 2.89)
	*AML & MDS*	4,142 (5%)	32.6%	824	1,352	1.64 (1.55, 1.73)
	*Hodgkin lymphoma*	1,240 (1%)	14.4%	247	178	0.72 (0.61, 0.83)
	**Solid tumors**					
	*NBL*	7,796 (9%)	31.3%	1,551	2,444	1.58 (1.51, 1.64)
	*Renal*	5,971 (7%)	22.9%	1,188	1,365	1.15 (1.09, 1.21)
	*STS*	5,028 (6%)	16.6%	1,001	836	0.84 (0.78, 0.89)
	**CNS tumors**					
	*EPN/choroid plexus*	1,768 (2%)	22.2%	352	393	1.12 (1.00, 1.23)
	*Glioma*, *all* ^c^	11,363 (14%)	3.1%	2,261	352	0.16 (0.14, 0.17)
	*MBL*	2,727 (3%)	17.1%	543	466	0.86 (0.78, 0.94)
**10–19 years**	**Hematologic malignancies**					
	*ALL & LyL*	10,312 (11%)	48.9%	2,052	5,044	2.46 (2.39, 2.53)
	*AML & MDS*	4,133 (4%)	29.3%	822	1,210	1.47 (1.39, 1.55)
	*Hodgkin lymphoma*	11,361 (12%)	13.2%	2,261	1,504	0.67 (0.63, 0.70)
	**Solid tumors**					
	*NBL*	599 (<1%)	17.9%	119	107	0.90 (0.73, 1.07)
	*Bone*	7,209 (8%)	19.4%	1,435	1,397	0.97 (0.92, 1.02)
	*STS*	7,208 (8%)	10.8%	1,434	780	0.54 (0.51, 0.58)
	**CNS tumors**					
	*EPN/choroid plexus*	861 (1%)	18.7%	171	161	0.94 (0.79, 1.08)
	*Glioma*, *all* ^c^	9,425 (10%)	3.5%	1,876	331	0.18 (0.16, 0.19)
	*MBL*	1,198 (1%)	25.0%	238	300	1.26 (1.12, 1.40)
**20–29 years**	**Hematologic malignancies**					
	*ALL & LyL*	4,719 (2%)	15.1%	939	714	0.76 (0.70, 0.82)
	*AML & MDS*	5,513 (2%)	3.7%	1,097	204	0.19 (0.16, 0.21)
	*Hodgkin lymphoma*	22,597 (9%)	1.0%	4,497	236	0.05 (0.05, 0.06)
	**Solid tumors**					
	*Bone*	3,905 (2%)	9.6%	777	375	0.48 (0.43, 0.53)
	*STS*	12,250 (5%)	1.6%	2,438	199	0.08 (0.07, 0.09)
	**CNS tumors**					
	*Glioma*, *all*^c^^b^	10,924 (5%)	0.4%	2,174	48	0.02 (0.02, 0.03)

^a^ Percentages do not sum to 100 given inclusion of only most prevalent pediatric/AYA malignancy diagnoses.

^b^ Using total percent enrollment of patients 0 to 19 years old (19.9%).

^c^ A large number of glioma diagnoses did not include grading information in SEER.

Higher enrollment than expected by SEER.

Lower enrollment than expected by SEER.

Abbreviations: SR, Standardized Ratio of Enrollment; CI, Confidence Interval; ALL, acute lymphoblastic leukemia; LyL, lymphoblastic lymphoma; NBL, neuroblastoma; STS, soft tissue sarcoma; CNS, central nervous system; EPN, ependymoma; MBL, medulloblastoma; SEER, Surveillance, Epidemiology, and End Results.

### Enrollment by age, race/ethnicity, and disease type

Enrollment was assessed in subgroups stratified by age, race/ethnicity, and disease type (Table 4). Across all race/ethnicities and major disease types, enrollment was strongly affected by age, with 5 to 9-year-olds having the highest relative enrollment, followed by 0 to 4-year-olds, then 10 to 14-year-olds, and 15 to 19-year-olds. American Indian/Alaskans were enrolled relatively less across all tumor types. Among patients age 0 to 9 years with hematologic malignancies, Whites were relatively overrepresented while enrollment of Blacks with hematologic malignancies was relatively reduced from expected as well as in relation to enrollment of Whites, Hispanics, and Asian/Pacific Islanders.

**Table 4:** COG enrollment stratified by disease type, age, and race, 2004–2015.

Disease Type	Age (years)	Race/Ethnicity ^a^				
		White	Black	Hispanic	Asian/ Pacific Islander	American Indian/Alaskan
		SR (95% CI) ^b^	SR (95% CI) ^b^	SR (95% CI) ^b^	SR (95% CI) ^b^	SR (95% CI) ^b^
		% Enrolled	% Enrolled	% Enrolled	% Enrolled	% Enrolled
**Hematologic malignancies**	**Total** **(0 to 29)**	0.99 (0.98, 1.01)	0.72 (0.69, 0.75)	1.15 (1.12, 1.18)	0.93 (0.87, 0.98)	**0.59 (0.49, 0.70)**
		19.8%	14.3%	22.9%	18.4%	**11.8%**
	**0 to 4**	2.34 (2.28, 2.40)	1.83 (1.69, 1.96)	1.87 (1.79, 1.94)	1.87 (1.69, 2.05)	**1.10 (0.80, 1.40)**
		46.6%	36.4%	37.2%	37.3%	**21.9%**
	**5 to 9**	2.38 (2.30, 2.46)	1.74 (1.59, 1.90)	2.17 (2.06, 2.27)	1.96 (1.72, 2.2)	**1.60 (1.07, 2.13)**
		47.4%	34.7%	43.1%	39.0%	**31.8%**
	**10 to 14**	1.33 (1.28, 1.39)	0.99 (0.89, 1.08)	1.30 (1.21, 1.38)	1.12 (0.94, 1.31)	**0.60 (0.32, 0.88)**
		26.5%	19.6%	25.9%	22.4%	**12.0%**
	**15 to 19**	0.98 (0.94, 1.02)	0.86 (0.78, 0.94)	1.17 (1.10, 1.25)	0.94 (0.79, 1.08)	**0.35 (0.14, 0.56)**
		19.5%	17.2%	23.4%	18.7%	**7.0%**
	**20 to 24**	0.20 (0.18, 0.21)	0.15 (0.12, 0.18)	0.25 (0.22, 0.28)	0.21 (0.15, 0.27)	**0.15 (0.03, 0.27)**
		3.9%	3.0%	4.9%	4.1%	**3.0%**
	**25 to 29**	0.02 (0.02, 0.03)	0.01 (0.01, 0.02)	0.04 (0.03, 0.05)	0.02 (0.00, 0.03)	**0** ^**c**^
		0.5%	0.3%	0.8%	0.3%	
**Solid tumors**	**Total** **(0 to 29)**	0.17 (016, 0.17)	0.25 (0.24, 0.27)	0.17 (0.17, 0.18)	0.15 (0.13, 0.17)	**0.09 (0.06, 0.11)**
		3.3%	5.0%	3.5%	3.0%	**1.7%**
	**0 to 4**	1.00 (0.96, 1.03)	0.85 (0.78, 0.93)	0.74 (0.69, 0.80)	0.77 (0.65, 0.89)	**0.35 (0.18, 0.52)**
		19.8%	17.0%	14.8%	15.3%	**7.0%**
	**5 to 9**	1.05 (0.98, 1.11)	0.94 (0.82, 1.06)	0.76 (0.66, 0.86)	0.90 (0.65, 1.15)	**0.56 (0.17, 0.94)**
		20.8%	18.7%	15.1%	17.9%	**11.1%**
	**10 to 14**	0.47 (0.44, 0.51)	0.50 (0.43, 0.58)	0.36 (0.32, 0.41)	0.35 (0.22, 0.46)	**0.28 (0.06, 0.50)**
		9.4%	10.0%	7.3%	6.9%	**5.6%**
	**15 to 19**	0.23 (0.21, 0.24)	0.39 (0.34, 0.44)	0.23 (0.20. 0.26)	0.18 (0.13, 0.24)	**0.13 (0.03, 0.23)**
		4.5%	7.7%	4.6%	3.6%	**2.7%**
	**20 to 24**	0.04 (0.03, 0.04)	0.06 (0.05, 0.08)	0.03 (0.03, 0.04)	0.03 (0.01, 0.04)	**0.01 (-0.01, 0.03)**
		0.7%	1.2%	0.7%	0.6%	**0.2%**
	**25 to 29**	0.01 (0.00, 0.01)	0.01 (0.00, 0.01)	0.00 (0.00, 0.00)	0.01 (0.00, 0.01)	**0** ^**c**^
		0.1%	0.1%	0.0%	0.1%	
**CNS tumors**	**Total** **(0 to 29)**	0.25 (0.24, 0.26)	0.29 (0.26, 0.33)	0.30 (0.27, 0.32)	0.26 (0.20, 0.31)	**0.15 (0.06, 0.24)**
		5.0%	5.9%	5.9%	5.1%	**3.0%**
	**0 to 4**	0.29 (0.26, 0.32)	0.28 (0.22, 0.35)	0.33 (0.27, 0.39)	0.26 (0.14, 0.37)	**0.19 (-0.02, 0.40)**
		5.8%	5.7%	6.6%	5.1%	**3.7%**
	**5 to 9**	0.42 (0.39, 0.46)	0.50 (0.40, 0.59)	0.44 (0.36, 0.51)	0.36 (0.22, 0.51)	**0.24 (-0.03, 0.50)**
		7.9%	9.9%	8.7%	7.2%	**4.7%**
	**10 to 14**	0.40 (0.36, 0.44)	0.31 (0.23, 0.39)	0.42 (0.34, 0.51)	0.43 (0.26, 0.61)	**0.12 (-0.11, 0.35)**
		7.9%	6.2%	8.4%	8.6%	**2.3%**
	**15 to 19**	0.31 (0.27, 0.34)	0.40 (0.30, 0.50)	0.34 (0.26, 0.43)	0.29 (0.15, 0.43)	**0.26 (-0.03, 0.30)**
		6.1%	8.0%	6.9%	5.8%	**5.2%**
	**20 to 24**	0.09 (0.07, 0.11)	0.12 (0.06, 0.18)	0.10 (0.05, 0.15)	0.14 (0.04, 0.24)	**0** ^**c**^
		1.8%	2.4%	2.0%	2.8%	
	**25 to 29**	0 ^c^	0 ^c^	0 ^c^	0.02 (-0.02, 0.05)	**0** ^**c**^
					**0.3%**	

^a^ 2+ races and unknown race/ethnicity patients were excluded given no corresponding US SEER data available.

^b^ Using total percent enrollment of patients 0 to 19 years old (19.9%).

^c^ Indicates that no patients of this subgroup enrolled into COG therapeutic trials.

Abbreviations: SR, Standardized Ratio of Enrollment; CI, Confidence Interval; CNS, Central Nervous System.

### Enrollment rate over time & assessment of available trials

**Fig 2:**
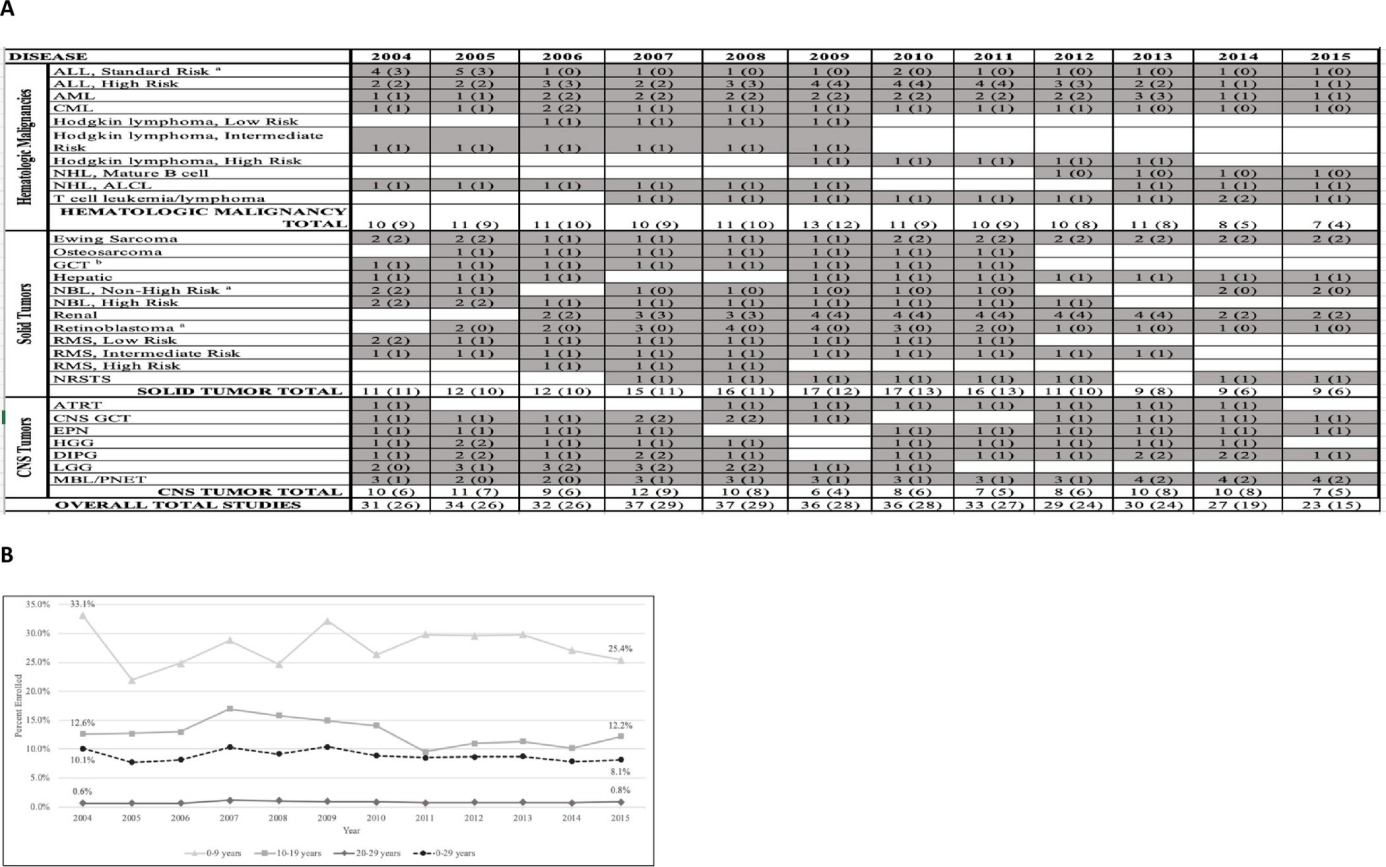
COG enrollment rates and available trials, 2004–2015. (A) Available Children’s Oncology Group (COG) trials for newly diagnosed patients by disease, 2004–2015* (B) Rate of COG enrollment by age and year. *Presented data is based upon “Opened for Entry” and “Study Closed” dates on the COG member website. Columns represent number of trials open at any time during that year, overall and in which upper age eligibility ≥ 18-years-old (in parenthesis). ^a^ Upper age limit for eligibility likely impacted by patient risk stratification definition and age population affected. ^b^ Trials for ≥ 18-year-old patient population limited to ovarian and extragonadal tumor only, upper age eligibility for testicular tumor < 15 years old. Abbreviations: ALL, acute lymphoblastic leukemia; AML, acute myeloid leukemia; CML, chronic myeloid leukemia; NHL, non-Hodgkin lymphoma; ALCL, anaplastic large cell lymphoma; GCT, germ cell tumor; RMS, rhabdomyosarcoma; NRSTS, non-rhabdomyosarcoma soft tissue sarcoma; CNS, central nervous system; ATRT, atypical teratoid rhabdoid tumor; EPN, ependymoma; HGG, high grade glioma; DIPG, diffuse intrinsic pontine glioma; LGG, low grade glioma; MBL, medulloblastoma; PNET, primitive neuroectodermal tumor.

An assessment of available COG trials during the study period for major disease types (A) as well as enrollment rates by year, stratified by age group (B) is depicted in Fig 2. The enrollment rate of the entire cohort declined over the study period (from 10.1% to 8.1%), with the largest rate of change present in the 0-9-year-old group (from 33.1% to 25.4%). The total number of available, upfront COG trials peaked in 2007–2008. Diseases with the most consistently available trials included ALL, AML, chronic myeloid leukemia, Ewing sarcoma, retinoblastoma, diffuse intrinsic pontine glioma, and MBL/peripheral neuroectodermal tumor. Diseases with the least consistency in annually available trials included Hodgkin lymphoma, mature B cell lymphoma, osteosarcoma, high risk rhabdomyosarcoma, and low grade glioma. The most commonly used upper age limits for eligibility were 21 (hematologic and CNS tumors) and 30 years old (solid tumors), though limits showed significant variation overall. Fig 2A also displays the total number of available trials in which the upper age eligibility was > 18 years old, accounting for 65–84% of available trials overall depending on year.

## Discussion

Proportional representation across demographic groups for trial enrollment is an important gauge of equitable access and is necessary for adequate generalizability of results. Prior reports of disparities in pediatric oncology outcomes by race/ethnicity [20, 21] and the suggestion of survival benefit with trial enrollment, [22–24] particularly among AYA patients known to be a susceptible population for health disparities, [25] further emphasize the necessity of ensuring proportional access to trials and identifying barriers to enrollment.

In our analysis, 19.9% of cancer cases from birth to 19 years old using SEER registry rates were enrolled onto upfront COG therapeutic trials between 2004 and 2015, an estimate that is reduced from the 26.8% COG enrollment rate reported by Lund *et al*. between 2000 and 2003.(9) Importantly, our study used identical methodology to Lund *et al*. with the exception of further including standardized ratios of enrollment for easier comparison across subgroups. Thus, although childhood cancer incidence has risen over several decades, [26] enrollment rates appear to be declining, from 40–70% reported in the 1990’s, [3, 8, 27] to approximately 20–25% in the 2000’s. Our analysis of trial availability during the study period evinced that the total number of broad, upfront COG trials peaked in 2007–2008 and then showed an overall decline from 2009 to 2015, though it should be noted that this analysis was limited to the use of opening and closure dates for each study, which may not accurately reflect periods of actual patient accrual. As cure rates have improved for common diagnoses such as standard risk ALL, trial development focus may have shifted toward high-risk subtypes or diagnoses with continued poor response. Certainly, the sample sizes necessary to show differences in outcome for high survival diseases make opening trials less feasible for those particular diagnoses, and the expectation that every diagnosis will have an available COG trial is unlikely to continue. On a global scale, the relatively low mutational burden of pediatric tumors [28] translates to a limited number of targets available for drug development overall. Further, the shift from histologic to molecular characterization to define trial eligibility may have also impacted the total number of available trials during this time period. Finally, a reduction in NCI funding over the study period and the complex approval process of investigational new agents may have also limited the ability to open new trials. Clearly, a continued emphasis on trial enrollment remains important to both improve cure rates for high-risk diagnoses and answer remaining questions within highly curable diagnoses, such as risk stratification and treatment de-intensification.

Age was the most notable factor affecting enrollment in our analysis, with younger patients consistently more represented across all diseases and races. Historically, enrollment for the AYA population has been below that of pediatric counterparts, [6, 8, 11–13] and in 2006 the NCI identified AYA patients as a distinct health disparity population. Several studies cite a lack of available trials as a contributor to reduced AYA enrollment, [6, 12, 29–31] and in our analysis of available COG trials many of the diseases with reduced availability were those that predominate in AYA patients, such as Hodgkin lymphoma and osteosarcoma. A large body of research has identified additional factors leading to reduced enrollment for AYA patients such as site of care, poor physician referral rates, suboptimal insurance, and psychosocial factors like informed consent concerns and lack of knowledge about trials. [12, 29, 32–37] The higher rate of AYA enrollment for ALL/LyL patients in our analysis likely stems from publications demonstrating superior outcomes for AYAs with ALL treated on pediatric compared to adult protocols. [38–41] Interestingly, particularly among the AYA age groups, males were shown to be slightly more represented than females; thus, females may represent a particularly vulnerable subgroup to health disparities that warrants further examination. Importantly, given this study was limited to evaluation of COG enrollment only and did not include adult cooperative group, consortia or institutional trials, we are likely underrepresenting total AYA enrollment to all trials available to this unique population. Additionally, the inclusion of “other” and “not otherwise specified” malignancies in our SEER cohort may have affected the interpretation of COG enrollment rates given that adult-predominant malignancies are included within these categories and COG would not have had open trials for those diagnoses. While we recognize the difficulty in drawing substantial conclusions for the AYA population based on these limitations, we feel there is significant utility in describing COG’s contribution to this population’s trial participation. As the largest pediatric and adolescent cooperative cancer research group in the world, assessing enrollment of AYA patients to COG trials effectively estimates the participation rate in US pediatric clinical trials for these patients, and this estimate can then serve as a comparator for later time points. COG has made efforts to expand eligibility criteria to improve trial access for AYA patients, though our analysis did identify variability in upper age limits for trial eligibility within malignancies common to this older cohort, with the continued exclusion of many AYA patients during the examined study period. Of course, many additional factors influence the “true” availability of a clinical trial to an individual patient (trial being open at local institution, physician’s decision to present the trial, patient meeting eligibility criteria) and the choice to enroll, particularly among the AYA population. Although the collaborative efforts between COG and the NCI’s National Clinical Trials Network (NCTN) has allowed adult cooperative group sites access to COG trials, further encouragement of additional NCTN groups to participate and a systematic movement of adult cooperative groups to lower their age eligibility is still needed.

Enrollment also varied by disease type, with increased enrollment in patients with hematologic malignancies compared to solid and CNS tumors. Similar findings were reported by Lund *et al*., and prior reports have reported approximately 55% enrollment for pediatric leukemia from 1990 to 2015, significantly more than has been estimated for overall pediatric enrollment. [2, 9, 10] This overrepresentation may stem from ongoing momentum achieved from historical successes in ALL, but it may also reflect the increased heterogeneity of diagnoses within solid and CNS tumors, with increased difficulty ensuring available trials for all disease types.

Finally, consistent with prior studies, [3, 8, 9] enrollment was grossly proportional across races and ethnicities as well as socioeconomic groups. This finding highlights the accessibility of COG trials to US patients and suggests that patients enrolled to COG trials are generally representative of the overall pediatric and AYA cancer population with regard to race/ethnicity and socioeconomic status. Of note, subtle variation did exist among races/ethnicities, with American Indian/Alaskan patients consistently enrolled less than expected across all disease types, and young (0-9-year-old) Black patients with hematologic malignancies enrolled relatively less than their White, Hispanic, and Asian/Pacific Islander counterparts.

The strengths of this study include the large sample size and extended time period evaluated, allowing for a modern, comprehensive evaluation of COG enrollment. Further, the expanded age range and inclusion of socioeconomic factors provide an expanded assessment of important populations known to be at risk for health disparities. The inherent limitations include an inability to control for patients who may not have required additional therapy following initial resection and/or radiation, and the inability to evaluate the “true” availability of trials for any given patient. Further, as discussed, the analysis was limited to COG only and does not include enrollment to adult cooperative group, pediatric consortia, or other locally available trials; thus, it may underestimate actual trial enrollment. Lastly, socioeconomic data was only available on a county rather than individual level, and therefore these variables may reflect the characteristics of highly populous or urban counties over less populated, rural areas. Of note, a recent analysis demonstrated no survival benefit for US childhood cancer patients living in urban versus rural areas, emphasizing that increased public health insurance access for children and the wide reach of COG to areas with fewer medical resources may be contributing to equitable trial access and outcomes, regardless of socioeconomic status. [42]

This study provides an updated and expanded assessment of pediatric and AYA COG trial participation. Future work should continue to [1] evaluate changes to enrollment rates over time, particularly as eligibility for trials becomes more consistently molecularly driven, [2] determine methods to more accurately evaluate the availability of trials undergoing active accrual, as opposed to using surrogate trial opening and closure dates, and [3] provide a more accurate assessment of AYA enrollment across all trials available to this patient population. The expansion of COG eligibility criteria to include young adults should broaden to include more disease types, systematic efforts to provide AYA patients access to adult cooperative group trials must occur, and the establishment of unified programs connecting pediatric and adult hospitals should continue at more sites to encourage AYA enrollment. [43] Trial enrollment has been a significant contributor to success seen in pediatric oncology, and a continued emphasis is required to provide treatment advances and improved outcomes for all.

## Supporting information

S1 Data (XLSX)
